# Segmental infantile hemangioma with milia: A case report

**DOI:** 10.1177/2050313X231164265

**Published:** 2023-04-14

**Authors:** Elena Pastukhova, Nordau Kanigsberg

**Affiliations:** 1 Faculty of Medicine, University of Ottawa, Ottawa, ON, Canada; 2 Division of Dermatology, The Ottawa Hospital, Ottawa, ON, Canada; 3 Division of Dermatology, Children’s Hospital of Eastern Ontario, Ottawa, ON, Canada

**Keywords:** Milia, milia-like cysts, infantile hemangioma, segmental hemangioma

## Abstract

Milia are common in children. They are small, keratinizing cysts that arise either as primary epidermoid cysts or secondary to other dermatoses, trauma or certain medications. In the paediatric population, milia are most frequently congenital and resolve spontaneously. Infantile hemangiomas are relatively common in neonates. They typically arise within the first few weeks of life, undergo a proliferative phase in the first 6 months, then begin to involute at around 12 months of age. After involution, residual skin changes may be observed, such as telangiectasia, fibrofatty tissue and redundant skin. However, there is a gap in the literature regarding concomitant milia and infantile hemangiomas. We report a case of a 5-months-old female who presented with a large, segmental infantile hemangioma of the posterior neck with milia.

## Introduction

Milia are superficial, keratinizing epidermoid cysts that are common in children. Histologically, the walls of a milium are composed of stratified squamous epithelium with a layer of granular cells that encase keratin. Milia can be classified into two major categories: primary and secondary.^1^ Primary milia arise from the sebaceous collar of vellus hair follicles and present as pearly, white-yellow papules. The most common type of primary milia is congenital milia, which are typically found on the face and present in 40%–50% of newborns without a sex predilection.^2^ Other causes of primary milia include certain genodermatoses, such as Bazex-Dupre-Christol syndrome.^1^ In comparison, secondary milia more commonly arise from eccrine ducts than from hair follicles and exhibit the same histopathologic findings as primary milia. Secondary milia are typically associated with other skin pathology, such as epidermolysis bullosa and other blistering dermatoses, as well as certain medications and skin trauma. Although milia are one of the most common, benign paediatric dermatoses, there is a paucity in data regarding their pathophysiology and significance in association with skin diseases.

Infantile hemangiomas (IHs) occur in about 4.5% of neonates and are more common in Caucasian females.^3^ It is hypothesized that the catalytic factor in IH development is transient hypoxia, which triggers increased angiogenesis in individuals with a genetic predisposition. Segmental hemangiomas are a subtype of IHs that cover an anatomic area, arise from one or more focal units, and measure ⩾ 5 cm.^4^ Cephalic segmental hemangiomas are more commonly associated with PHACES syndrome (posterior fossa malformations, hemangioma, arterial anomalies, coarcation of the aorta/cardiac defects, eye abnormalities, sternal malformations).^5^ Thus, prompt assessment and intervention are necessary.

Despite both milia and hemangiomas being relatively common dermatoses in children, there has only been one case report describing congenital hemangiomas with milia-like cysts.^6^ There have been no reported cases of milia arising in IHs. As such, the prevalence and significance, if any, of these comorbid conditions are unknown. Herein, we present an unusual case of a 5-months-old female who developed multiple milia overlying a segmental IH.

## Case report

A 5-months-old female with Fitzpatrick I skin presented with four vascular tumours on the posterior neck that were first noted within the first few weeks of life. The tumours exhibited initial rapid growth over a 4-week period. They restricted the patient’s neck movement. She did not have any neurological symptoms, such as seizures or abnormal tone. She had an episode of apnea lasting 10 s in the first week of life and wheezing until she reached 3.6 kg in weight. The patient was delivered, weighing 2.5 kg, via emergency Caesarean section at 38 weeks due to failure of labour progression and small-for-gestational age. She is otherwise healthy and has met normal developmental milestones.

**Figure 1.**
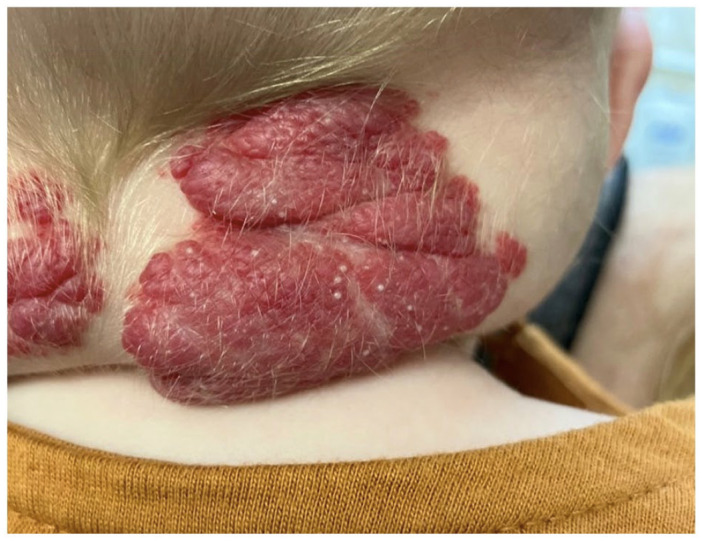
On the right posterior neck, there is a 4 × 5 cm exophytic tumour with an overlying red-violaceous plaque and multiple pearly, white papules 1–3 mm in size.

On examination of the posterior neck, there was a band of four 4–5 cm tumours with overlying red-violaceous discolouration. On the right lateral nodule, there were multiple white papules 1–3 mm in size, consistent with milia (Figure 1). Superior to the umbilicus, there is a 0.4 cm round, exophytic red-violaceous papule. No ulceration was present on any of the lesions. No sternal or umbilical raphe was noted. Due to concerns for PHACES syndrome, a magnetic resonance imaging (MRI) of the head, spine and thorax was performed, which was remarkable for four superficial and deep lesions involving the posterior neck, bilateral carotid spaces and extending to the prevertebral/retropharyngeal space. There was no evidence of posterior fossa abnormalities and no anomalies of the aortic arch. There was foetal origin of bilateral cerebral arteries with some tortuosity, but no evidence of aneurysm. Cardiological assessment was unremarkable, with normal ECG and no structural or functional abnormalities appreciated on echocardiography.

The patient was started on propranolol 0.5 mg/kg/day and was titrated up to 2.5 mg/kg/day with follow-ups every 2 weeks. She has tolerated the treatment well with no reported side effects. Improvement in the size, colour and texture of all hemangiomas has been noted. On the most recent visit, the milia have completely resolved.

## Discussion

Limited studies exist describing hemangiomas with milia. A recent cross-sectional study of 153 patients with segmental IHs did not identify secondary morphological features, such as milia.^6^ One study described 3 × 4 cm congenital superficial hemangiomas with overlying milia-like cysts located on the scalp and back of a 4-months-old male.^7^ The authors theorized the finding to represent either a hamartomatous structure or be secondary to the hemangiomatous disruption of developing hair follicles. Histologically, they noted keratinizing cysts lined with stratified squamous epithelium and surrounded by immature hair follicles. Similar to our patient, the milia-like cysts spontaneously resolved as the hemangiomas gradually regressed. Furthermore, it is useful to differentiate milia from pustules, as reports of pustules in congenital hemangiomas have been documented and theorized to represent tissue necrosis secondary to rapid involution.^8^

Due the relatively benign nature of both conditions, reporting bias may impact the prevalence of concomitant milia and hemangiomas in the literature. We hypothesize that the presence of milia represents a disruption of eccrine glands and/or hair follicles due to the growth and large size of the segmental IH. In addition, it is plausible that friction on posterior neck may have traumatized the skin, leading to milia. Interestingly, patient’s periumbilical IH did not have milia, which may support the skin trauma hypothesis. We hope the present case report may provide clinicians and patients with reassurance of this rare coexistence of these two common conditions.
